# Purinergic interplay between erythrocytes and platelets in diabetes-associated vascular dysfunction

**DOI:** 10.1007/s11302-021-09807-5

**Published:** 2021-08-19

**Authors:** Zhichao Zhou

**Affiliations:** Division of Cardiology, Department of Medicine Solna, Karolinska Institutet, Karolinska University Hospital, Stockholm, Sweden

**Keywords:** ATP, Circulation, Diabetes, Platelet, Purinergic receptor, Red blood cell

## Abstract

Cardiovascular complications in diabetes are the leading causes for high morbidity and mortality. It has been shown that alteration of purinergic signaling contributes to diabetes-associated cardiovascular complications. Red blood cells (RBCs) and platelets play a fundamental role in regulation of oxygen transport and hemostasis, respectively. Of note, these cells undergo purinergic dysfunction in diabetes. Recent studies have established a novel function of RBCs as disease mediators for the development of endothelial dysfunction in type 2 diabetes (T2D). RBC-released ATP is defective in T2D, which has implication for induction of vascular dysfunction by dysregulating purinergic signaling. Platelets are hyperactive in diabetes. ADP-mediated P2Y_1_ and P2Y_12_ receptor activation contributes to platelet aggregation and targeting P2Y receptors particularly P2Y_12_ receptor in platelets is effective for the treatment of cardiovascular events. In contrast to other P2Y_12_ receptor antagonists, platelet-targeting drug ticagrelor has potential to initiate purinergic signaling in RBCs for the beneficial cardiovascular outcomes. It is increasingly clear that altered vascular purinergic signaling mediated by various nucleotides and nucleoside contributes to diabetes-associated vascular dysfunction. However, the contribution of complex purinergic networks between RBCs and platelets to the vascular dysfunction in diabetes remains unclear. This study discusses the possible interplay of RBCs and platelets via the purinergic network for diabetes-associated vascular dysfunction.

## Introduction

Diabetes is an important risk factor for the development of a variety of cardiovascular diseases including atherosclerosis and ischemic heart disease. The vascular complications associated with diabetes are the leading causes for high morbidity and mortality worldwide [1, 2]. Vascular dysfunction plays a crucial role in the etiology of diabetes-induced vascular complications. This is characterized by imbalances between vasoconstrictor/inflammatory factors such as reactive oxygen species (ROS) or signaling pathways such as nucleot(s)ide-mediated vasoconstrictor purinergic signaling and vasodilator/anti-inflammatory factors such as nitric oxide (NO) or signaling pathways such as nucleot(s)ide-mediated vasodilator purinergic signaling [2]. These substances are released and/or initiated from the cardiovascular wall as well as circulating cells including red blood cells (RBCs) and platelets. The vasoconstrictor/inflammatory net effects of those substances acting on the vascular wall eventually result in vascular dysfunction [1]. Both purinergic P1 and P2 receptors are ubiquitously expressed in endothelial cells and smooth muscle cells in the vasculature [2]. It is increasingly clear that altered vascular purinergic signaling mediated by various nucleotides (e.g., ATP and ADP) and nucleoside (adenosine) significantly contributes to diabetes-associated vascular complications [2–5].

RBCs and platelets are the most abundant cells in the circulation, both of which play a fundamental role in cardiovascular homeostasis, due to their diverse functions including gas transportation, hemostasis, thrombosis, coagulation, and vascular regulation [1, 6]. Emerging studies have shown that RBCs act as disease mediators for the development of endothelial dysfunction in type 2 diabetes (T2D) [1, 7–10]. RBCs serve as ATP pool and release of ATP in response to low oxygen tension plays a crucial role in the regulation of tissue perfusion [11]. Of note, the release of ATP from RBCs in diabetes is defective, which results in less vasodilation in isolated muscle arterioles [12]. Of further interest, a recent study demonstrated that altered vascular purinergic signaling is involved in endothelial dysfunction induced by RBCs from patients with T2D [13]. Platelets are hyperactive in diabetes, which could further promote endothelial dysfunction and contribute to enhanced risk of development of atherothrombotic disease in various vasculatures including coronary and cerebral arteries [14, 15]. One of the important mechanisms of platelet activation is due to ADP-activated purinergic P2Y_1_ and P2Y_12_ receptors [14]. Therefore, targeting these receptors in particular P2Y_12_ receptors have been applied widely in the clinic to cope with the cardiovascular/thrombotic event [16, 17].

Given both RBCs and platelets of diabetes origin induce endothelial dysfunction and the significant involvement of purinergic signaling in both cell types, whether there is interplay between RBCs and platelets via purinergic signaling (de)activating vascular purinergic signaling for the induction of diabetes-associated vascular complications remains incompletely understood. The present study discusses complex purinergic networks as possible links for an interplay between RBCs and platelets for the development of vascular dysfunction in diabetes.

## RBC and purinergic signaling in diabetes

RBCs play a fundamental role in cardiovascular homeostasis because of their contribution to vascular function and integrity. RBCs become dysfunctional in diabetes, as evidenced by reduced NO bioactivity, enhanced oxidative stress, and ATP-mediated altered purinergic signaling [1, 8]. Such alterations may subsequently affect the vascular function and induce cardiovascular complications. Recent studies have revealed a novel function of RBCs as disease mediators for the development of T2D-associated endothelial dysfunction. It has been shown that RBCs from patients and rodents with T2D induced endothelial dysfunction and exacerbated cardiac ischemia–reperfusion injury [1, 7, 13, 18]. Interestingly, the detrimental effect of RBCs on endothelial function seems not to be solely induced by hyperglycemia, as evidenced by that RBCs of T2D patients with improved glycemic control could not attenuate endothelial injury [9]. The mechanisms underlying this potentially important function of RBCs for the development of cardiovascular dysfunction in T2D remain incompletely elucidated and warrant further investigations.

**Fig. 1:**
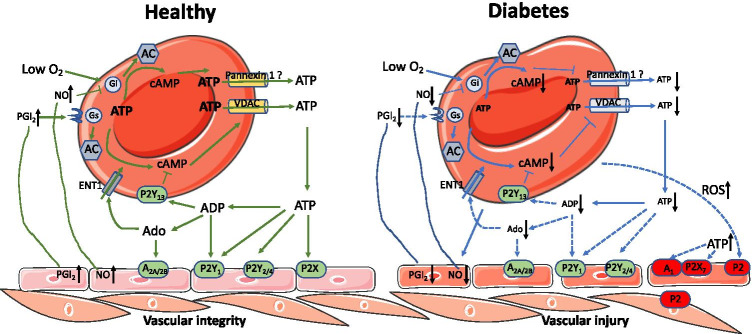
Red blood cells (RBCs) and purinergic signaling in diabetes. ATP is released from RBCs via voltage-dependent anion channels and likely pannexin 1. ATP can activate various P2X, P2Y_1_, P2Y_2_, and P2Y_4_ receptors on the endothelium leading to nitric oxide (NO) and prostacycline (PGI_2_) productions. ATP is degraded to ADP acting on P2Y_1_ on the endothelium. The further degradation product adenosine (Ado) activates A_2A_ and A_2B_ on the vasculature leading to vasodilation. NO and PGI_2_ interact with RBCs to inhibit hypoxia-induced ATP release and stimulate PGI_2_ receptor-mediated ATP release, respectively. ADP activates P2Y_13_ receptors in RBCs having negative feedback effect on ATP release. Ado is taken up by equilibrative nucleoside transporter 1 (ENT1) in RBCs for its metabolism. In diabetes, ATP release from RBCs is impaired. Accordingly, the downregulated ATP and its degradation products affect the corresponding purinergic receptors leading to less vasodilation. Moreover, endothelial dysfunction induced by RBCs of diabetes is through alteration of vascular purinergic P2X_7_ and A_1_ receptors. Increased reactive oxygen species (ROS) is speculated to activate vascular ATP release for the activation of vasoconstrictor P2X_7_ and A_1_ receptors. The effects of NO and PGI_2_ as well as ADP-mediated P2Y_13_ activation on ATP release from the RBCs in diabetes remain to be determined. Solid lines indicate established pathways, while dash lines indicate the pathways need to be proved in the future studies. Black arrows toward up indicate increasing effects, while black arrows toward down indicate decreasing effects. Question mark indicates the role that is currently under controversy

RBCs can release ATP in response to physiological stimuli [11, 12]. This is evidenced in studies that there is substantial ATP release from human RBCs in response to low oxygen tension without detectable hemolysis [12, 19]. In contrast, it is suggested that ATP release from RBCs occurs during cell lysis, which may be physiologically relevant during exercise and hypoxia when intravascular hemolysis of senescent cells is increased [20]. The ATP release in response to low oxygen tension requires increases in cAMP induced by activation of heterotrimeric G (Gi) protein-stimulated adenylyl cyclase (AC) [11]. ATP is suggested to be transported via pannexin-1 channels [21] (Fig. 1). The role of pannexin-1 in ATP release may need further validations, as one study using pannexin-1 knockout mice shows an unlikely involvement [22]. Once released, ATP can be degraded to ADP and adenosine by various nucleotidases. These adenine nucleotides activate their corresponding purinergic receptors to regulate blood flow and tissue perfusion [2, 11, 23]. RBC-derived ATP can bind to vasodilator P2 receptors on the endothelium to generate vasodilators and anti-inflammatory factors [e.g., NO and prostacyclin (PGI_2_)] for subsequent vasodilation [11]. Interestingly, NO and PGI_2_ interact with RBCs to inhibit hypoxia-induced ATP release and stimulate PGI_2_ receptor-mediated ATP release via voltage-dependent anion channels, respectively [8, 11]. In addition to ATP release, RBCs express many purinergic receptors including both P1 and P2 receptors [24, 25]. A_2B_, P2Y_1_, P2Y_12_, and P2Y_13_ receptors are commonly expressed in RBCs, and the P2Y_13_ receptors are most abundantly expressed in both RBCs and reticulocytes [24, 25]. ADP can activate P2Y_13_ receptors in RBCs to affect cAMP levels leading to inhibition of ATP release [24]. In addition to vasodilation mediated by adenosine acting on adenosine receptors, adenosine is taken up by RBCs via equilibrative nucleoside transporter 1 (ENT1) for subsequent metabolism [1, 5, 26] (Fig. 1).

The release of ATP from RBCs is defective in diabetic patients [11, 12]. One study suggests that such impairment is associated with more senescent RBCs in diabetes [27]. As RBC P2Y_13_ receptors exert negative feedback effect for ATP release [24], it is of interest to investigate whether this receptor is involved in impairment of the ATP release in diabetes. Of further importance, the impaired ATP release from RBCs is associated with less vasodilation in arteries incubated with RBCs from patients with T2D [12] (Fig. 1). One proposed mechanism is pointed to less activation of ATP/ADP-mediated P2Y_1_ receptors and subsequent less NO and PGI_2_ production [2, 11]. It is possible that less PGI_2_ production in diabetes further downregulates PGI_2_ receptor-mediated ATP release in RBCs. The negative feedback effect of less NO generation in diabetes on ATP release in RBCs remains unclear, however. On the other hand, the impaired vasodilation could be partially attributed to upregulation of vasoconstrictor purinergic receptors activated by RBCs of T2D. This is supported by a recent study showing that vasoconstrictor A_1_ and P2X_7_ receptors are involved in endothelial dysfunction induced by RBCs from T2D patients [13] (Fig. 1). However, activation of those vascular purinergic receptors is unlikely due to a direct stimulus of the impaired ATP release from RBCs. The authors speculated that increased ROS formation derived from RBCs in diabetes may stimulate ATP release in other (endothelial) cells than RBCs to activate P2X_7_ receptors in endothelium and the degraded product adenosine could then activate the vasoconstrictor A_1_ receptors [13]. More studies are needed to elucidate the key purinergic signaling transmitted between the RBCs and vascular wall.

## Platelet and purinergic signaling in diabetes

Platelets play a crucial role in the regulation of hemostasis, thrombosis, coagulation, and vascular function [6, 28]. A close interaction with vascular wall has been well recognized that platelet activation and the local initiation of the coagulation cascade that lead to thrombus formation are usually observed at the site of vascular injury [6, 14]. Of note, platelets are hyperactive in diabetes, as is reflected by several dysregulated signaling pathways (e.g., hyperglycemia and oxidative stress) in platelets that lead to an increased tendency to activate and aggregate in response to even a low-grade stimulus [14, 15]. This leads to the pathology by not only promoting thrombus formation but also causing microvascular embolization and endothelial dysfunction that accelerate progression of local vascular damage [14, 29]. This process significantly contributes to diabetes-associated cardiovascular events such as myocardial infarction and stroke [30].

**Fig. 2:**
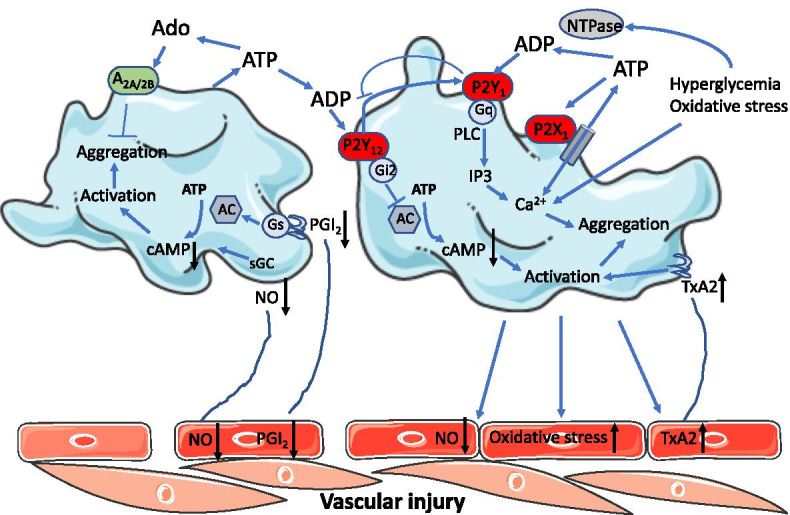
Platelets and purinergic signaling in diabetes. Platelets release ATP upon stimulation by hyperglycemia or oxidative stress via pannexin 1, which is subsequently degraded to ADP and adenosine (Ado). ATP and ADP activate P2X_1_ and P2Y_1_ receptors leading to increase in calcium concentration and platelet aggregation. ADP activates P2Y_12_ receptors resulting in platelet activation and stabilization of platelet aggregation. Activation of P2Y_12_ receptors positively regulates P2Y_1_ receptor-mediated action, while activation of P2Y_1_ receptors negatively regulates the effect of P2Y_12_ receptors. In contrast, Ado exerts inhibitory effects on platelet activation via A_2A_ and A_2B_ receptors. Activity of nucleotidase such as nucleoside triphosphate diphosphohydrolase (NTPase), an enzyme that hydrolyzes ATP and ADP, is elevated. Decreased productions of nitric oxide (NO) and prostacyclin (PGI_2_) in diabetes stimulate platelet activation. On the other hand, activated platelets further promote vascular injury by increasing oxidative stress and thromboxane A2 (TxA2) production and decreasing NO bioavailability. Black arrows toward up indicate increasing effects, while black arrows toward down indicate decreasing effects

One of the mechanisms underlying platelet activation has been shown to be due to the involvement of ADP-mediated purinergic signaling [6, 31] (Fig. 2). Several purinergic receptors including A_2A_, A_2B_, P2X_1_, P2Y_1_, and P2Y_12_ receptors are expressed and functional in human platelets [26, 32]. Activation of pannexin-1 in platelets via collagen leads to an exchange of calcium and ATP [30]. Extracellular ATP activates P2X_1_ receptors leading to platelet shape change through extracellular calcium influx [4, 31]. ATP is subsequently degraded to ADP and adenosine [31]. ADP exerts a positive feedback on platelet reactivity. Activation of the P2Y_1_ receptor in response to ADP leads to shape change and initiation of platelet aggregation. The activation of P2Y_1_ receptors stimulates phospholipase C (PLC)-inositol triphosphate (IP3) axis leading to mobilization of intracellular calcium [31]. ADP-activated P2Y_1_ receptors also exert synergistic effect to potentiate the ATP-P2X_1_ receptor-mediated action [4, 31]. In addition to activating P2Y_1_ receptors, ADP activates Gi-coupled P2Y_12_ receptors resulting in stabilization of platelet aggregation [31]. The Gαi2 subunit inhibits AC, thereby reducing cAMP levels. Although inhibition of AC is a key feature of platelet activation by ADP, this process has no causal relationship to platelet aggregation [33]. There is a complex signaling interaction between P2Y_1_ and P2Y_12_ receptors. Activation of P2Y_12_ receptors positively regulates P2Y_1_ receptor-mediated action, while activation of P2Y_1_ receptors negatively regulates the effect of P2Y_12_ receptors [34]. In contrast, adenosine could exert inhibitory effects on platelet activation via A_2A_ and A_2B_ receptors resulting in increased cAMP formation mainly during tissue injury and inflammation [26, 31] (Fig. 2).

Accumulative evidence has shown an alteration of purinergic components in platelets in diabetes (Fig. 2). Platelet ATP and ADP levels from patients with diabetes were higher than in platelets from healthy subjects [35]. Stimulation with thrombin in those platelets caused greater release of ATP and ADP than in the healthy group [35]. Significant correlations between platelet ATP/ADP and platelet activities have been found in diabetic patients [35]. There are also more abundantly expressed P2Y_1_ and P2Y_12_ receptors in platelets in diabetes [15]. Nucleoside triphosphate diphosphohydrolase (NTPDase), an enzyme that hydrolyzes ATP and ADP, was found to be elevated in platelet-rich plasma preparations from diabetic patients and rats [36–41]. Studies on the role for 5’-nucleotidase that degrades AMP to adenosine are not consistent. The enzyme activity in diabetes was observed to be decreased [36], unaltered [41], or increased [37–40]. Adenosine deaminase was found to be either increased or unaltered in platelets of diabetic rats [36, 38]. There is also evidence showing hyperglycemia has direct impact on ATP, ADP, and AMP hydrolysis in T2D patients [42]. In addition to the platelet activation upon vascular injury, activated platelets in diabetes have shown to cause endothelial dysfunction in healthy vasculature by increasing vascular oxidative stress, thromboxane A_2_ production, and reducing NO bioavailability [29], suggesting a vicious circle for promoting vascular injury (Fig. 2). However, the functional implications of the altered adenine nucleotides and enzymes in platelets for the diabetes-associated vascular complications remain to be determined.

## Interplay between RBCs and platelets

**Fig. 3:**
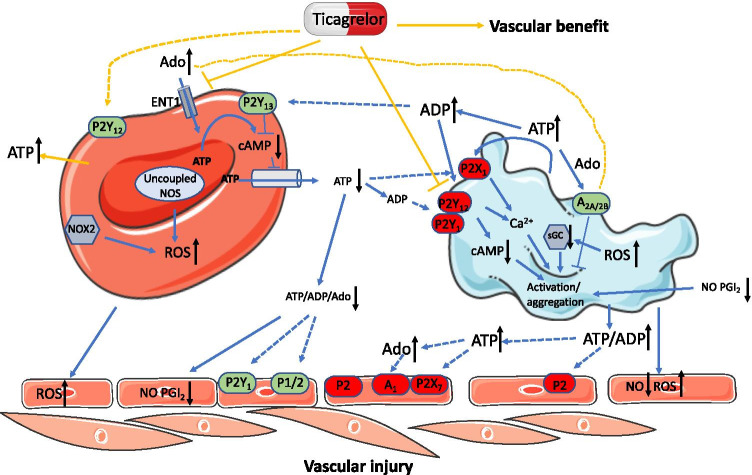
Interplay between RBCs and platelets in diabetes. ATP release from RBCs in diabetes is defective, which together with its degradation products induces less vasodilation. Platelets become sensitive to stimuli in diabetes; the less amount of ATP and ADP derived from RBCs may still activate platelets via P2X_1_, P2Y_1_, and P2Y_12_ receptors. Decreased bioavailability of nitric oxide (NO) and prostacyclin (PGI_2_) and increased reactive oxygen species (ROS) from endothelial cells as well as RBCs could promote platelet activation. Enhanced platelet-derived purine/adenosine (Ado) may activate vasoconstrictor purinergic receptors (e.g., A_1_ and P2X_7_) upregulated by dysfunctional RBCs resulting in vascular injury. Whether there is a crosstalk between enhanced platelet-derived purine/Ado and P2Y_13_ receptors in RBCs remains unclear. Ticagrelor has been thought to mainly target P2Y_12_ receptors in platelets for platelet aggregation. The pleiotropic cardiovascular effects by ticagrelor in diabetes are proposed to be via its additional actions on ATP release and inhibition of Ado uptake from RBCs. Whether ticagrelor directly activates P2Y_12_ receptor in RBCs remains to be determined. Solid lines indicate established pathways, while dash lines indicate the pathways need to be proved in the future studies. Black arrows toward up indicate increasing effects, while black arrows toward down indicate decreasing effects. Yellow lines indicate the effects of ticagrelor on purinergic signaling between erythrocytes and platelets in diabetes

Platelets have been thought to be the first cells being recruited to the site of vascular injury. However, this was challenged by a study showing that RBCs were the first cells adhering to the injured endothelium/vascular wall followed by recruitment of platelets [43]. Both RBCs and platelets of T2D origin induce vascular injury through increased formation of ROS, decreased NO bioavailability, and possibly altering purinergic signaling [7, 29]. However, the current knowledge regarding the purinergic signaling regulating a functional interaction between RBCs and platelets in the circulation in diabetes is sparse. NO and PGI_2_ have been established as potent inhibitors for platelet aggregation [6, 28]. Decreased NO bioactivity and PGI_2_ from endothelial cells and RBCs (likely via impaired ATP release) in diabetes further promote platelet activation (Fig. 3). Platelets are hyperactive in diabetes, which are more sensitive to activation even by a weak stimulation of purines [15]. Despite less ATP/ADP released from RBCs, it can be speculated that platelets could still be activated by impaired RBC-derived ATP and subsequent less degradation product of ADP in diabetes (Fig. 3). In contrast to impairment of ATP release from RBCs, platelet-derived ATP-ADP signaling is enhanced in diabetes, which exerts positive feedback on P2Y_1_ and P2Y_12_ receptors on platelets promoting platelet activation and aggregation [31]. As mentioned above, ATP release from RBCs is defective in diabetes, which results in less vasodilation [2]. It is unlikely that enhanced platelet-derived ATP-ADP signaling exerts any compensatory or beneficial effects on vascular function, as the overall effect of platelets isolated from diabetic animals is to induce endothelial dysfunction [29]. Instead, enhanced platelet-derived ATP in diabetes may stimulate vascular vasoconstrictor purinergic receptors in the circulation that are upregulated by the dysfunctional RBCs [13] (Fig. 3). More studies are needed to investigate the contribution of enhanced platelet-derived ATP/ADP to the activation of vascular purinergic receptors in diabetes. Moreover, how platelet-derived ADP in diabetes affects P2Y_13_ receptors on RBCs for the subsequent ATP release remains unknown and is of interest for further investigations.

Several P2Y_12_ receptor antagonists like clopidogrel and ticagrelor have been developed and are commonly prescribed to target ADP-mediated P2Y_12_ receptor activation in platelets for the treatment of thrombosis, stroke, and myocardial infarction in millions of patients with/without diabetes [16]. In addition to targeting platelets, ticagrelor is the only drug influencing RBCs [26], which may have a great potential to be involved in the interplay between RBCs and platelets. Indeed, ticagrelor can induce substantial amount of ATP release from RBCs by changing the membrane potential of RBCs, which are inhibited by anion transporter inhibitors 5-nitro-2-(3-phenylpropylamino)benzoic acid (NPPB) and probenecid [44]. Although P2Y_12_ receptors are present in the human RBCs [24], it remains unknown whether ticagrelor could activate P2Y_12_ receptors in RBCs for the ATP release. Of further interest, ticagrelor can target RBC ENT1 to inhibit adenosine uptake by RBCs, thereby increasing concentration of adenosine in the circulation [45]. Through these two pathways, ticagrelor-induced ATP release from RBCs may compensate for the impairment of ATP release from RBCs in T2D, thereby attenuating diabetes-associated vascular complications [26]. On the other hand, ticagrelor-inhibited adenosine uptake may counteract the ADP-mediated platelet activation via activation of A_2A_ and A_2B_ receptors [46] (Fig. 3). This hypothesis may increase the insights into understanding the potential mechanisms underlying the pleiotropic cardiovascular effects by ticagrelor in diabetes. These beneficial effects include anti-inflammation, increase in NO bioavailability, improvement in endothelial function, and decrease in major adverse cardiovascular event [47–49]. Given the abundance of RBCs and platelets and the active purinergic communication in the circulation, the RBCs and platelets may serve as potential therapeutic targets for the treatment of diabetes-associated vascular complications. The fact that the concentrations of RBCs exceed those of platelets in the circulation may require a higher dose of the future pharmacological agent to achieve an effective effect on RBCs.

## Conclusion

Both RBC- and platelet-derived purinergic signaling play a significant role in the regulation of vascular function in diabetes. There are complex purinergic networks communicating with each other between RBCs and platelets in the vasculature. However, the contribution of purinergic (in)activation derived from dysfunctional RBCs and platelets to the vascular dysfunction in diabetes remains unclear. Experimental studies to evaluate the proposed interaction of purinergic signaling between RBCs and platelets for diabetes-associated vascular dysfunction are warranted. A better understanding of the purinergic communication between these cells will enhance their potentials as targets for the treatment of diabetes-associated vascular complications.

## Data Availability

Not applicable.
